# Supplementary material for: Influence of pathway topology and functional class on the molecular evolution of human metabolic genes
Source: PLoS One. 2018 Dec 14;13(12):e0208782. doi: 10.1371/journal.pone.0208782 (PMC6294346; doi:10.1371/journal.pone.0208782)
Supplement: S1 Table — (DOC) [file pone.0208782.s001.doc]

| **S1 Table:** List of the 310 pathways, their codes, common names and their classifications. | | |  |
| --- | --- | --- | --- |
| **PATHWAY CODE** | **PATHWAY COMMON NAME** | **ONTOLOGY-BASED** | **COMPOUND-BASED** |
|  |  | **CLASSIFICATION** | **CLASSIFICATION** |
| 2PHENDEG-PWY | phenylethylamine degradation I | Degradation/Utilization/Assimilation | AminoAcid |
| ADENOSYLHOMOCYSCAT-PWY | methionine salvage | Biosynthesis | AminoAcid |
| ALANINE-DEG3-PWY | alanine biosynthesis/degradation | Degradation/Utilization/Assimilation | AminoAcid |
| ARGININE-SYN4-PWY | ornithine de novo biosynthesis | Biosynthesis | AminoAcid |
| ARGSPECAT-PWY | spermine biosynthesis | Biosynthesis | AminoAcid |
| ASPARAGINE-BIOSYNTHESIS | asparagine biosynthesis | Biosynthesis | AminoAcid |
| ASPARAGINE-DEG1-PWY | asparagine degradation | Degradation/Utilization/Assimilation | AminoAcid |
| ASPARTATESYN-PWY | aspartate biosynthesis | Biosynthesis | AminoAcid |
| BETA-ALA-DEGRADATION-I-PWY | &beta;-alanine degradation | Degradation/Utilization/Assimilation | AminoAcid |
| BSUBPOLYAMSYN-PWY | spermidine biosynthesis | Biosynthesis | AminoAcid |
| CYSTEINE-DEG-PWY | L-cysteine degradation I | Degradation/Utilization/Assimilation | AminoAcid |
| GLNSYN-PWY | glutamine biosynthesis | Biosynthesis | AminoAcid |
| GLUDEG-I-PWY | GABA shunt | Degradation/Utilization/Assimilation | AminoAcid |
| GLUTAMATE-SYN2-PWY | glutamate biosynthesis/degradation | Biosynthesis | AminoAcid |
| GLUTAMINDEG-PWY | glutamine degradation/glutamate biosynthesis | Degradation/Utilization/Assimilation | AminoAcid |
| GLUTATHIONESYN-PWY | glutathione biosynthesis | Biosynthesis | AminoAcid |
| GLYCLEAV-PWY | glycine cleavage | Degradation/Utilization/Assimilation | AminoAcid |
| GLYSYN-ALA-PWY | glycine biosynthesis | Biosynthesis | AminoAcid |
| GLYSYN-PWY | glycine/serine biosynthesis | Biosynthesis | AminoAcid |
| HOMOCYSDEGR-PWY | cysteine biosynthesis/homocysteine degradation | Degradation/Utilization/Assimilation | AminoAcid |
|  | (trans-sulfuration) |  |  |
| HYDROXYPRODEG-PWY | 4-hydroxyproline degradation | Degradation/Utilization/Assimilation | AminoAcid |
| ILEUDEG-PWY | isoleucine degradation | Degradation/Utilization/Assimilation | AminoAcid |
| LEU-DEG2-PWY | leucine degradation | Degradation/Utilization/Assimilation | AminoAcid |
| LYSINE-DEG1-PWY | lysine degradation I (saccharopine pathway) | Degradation/Utilization/Assimilation | AminoAcid |
| METHIONINE-DEG1-PWY | methionine degradation | Degradation/Utilization/Assimilation | AminoAcid |
| PHENYLALANINE-DEG1-PWY | phenylalanine degradation/tyrosine biosynthesis | Degradation/Utilization/Assimilation | AminoAcid |
| PROSYN-PWY | proline biosynthesis | Biosynthesis | AminoAcid |
| PROUT-PWY | proline degradation | Degradation/Utilization/Assimilation | AminoAcid |
| PWY-0 | putrescine degradation III | Degradation/Utilization/Assimilation | AminoAcid |
| PWY0-1305 | glutamate dependent acid resistance | Detoxification | AminoAcid |
| PWY-3661-1 | glycine betaine degradation | Degradation/Utilization/Assimilation | AminoAcid |
| PWY-40 | putrescine biosynthesis II | Biosynthesis | AminoAcid |
| PWY-4041 | &gamma;-glutamyl cycle | Superpathways | AminoAcid |
| PWY-4061 | glutathione-mediated detoxification | Detoxification | AminoAcid |

| PWY-4081 | glutathione redox reactions I | Biosynthesis | AminoAcid |
| --- | --- | --- | --- |
| PWY-46 | putrescine biosynthesis I | Biosynthesis | AminoAcid |
| PWY-4921 | protein citrullination | Biosynthesis | AminoAcid |
| PWY-4983 | citrulline-nitric oxide cycle | Degradation/Utilization/Assimilation | AminoAcid |
| PWY-4984 | urea cycle | Degradation/Utilization/Assimilation | AminoAcid |
| PWY-5030 | histidine degradation | Degradation/Utilization/Assimilation | AminoAcid |
| PWY-5046 | 2-oxoisovalerate decarboxylation to isobutanoyl- | Generation of Precursor Metabolites | AminoAcid |
|  | CoA | and Energy |  |
| PWY-5177 | glutaryl-CoA degradation | Degradation/Utilization/Assimilation | AminoAcid |
| PWY-5326 | sulfite oxidation | Degradation/Utilization/Assimilation | AminoAcid |
| PWY-5328 | superpathway of methionine degradation | Degradation/Utilization/Assimilation | AminoAcid |
| PWY-5329 | L-cysteine degradation II | Degradation/Utilization/Assimilation | AminoAcid |
| PWY-5331 | taurine biosynthesis | Biosynthesis | AminoAcid |
| PWY-5340 | sulfate activation for sulfonation | Degradation/Utilization/Assimilation | AminoAcid |
| PWY-5350 | thiosulfate disproportionation III (rhodanese) | Degradation/Utilization/Assimilation | AminoAcid |
| PWY-5651 | tryptophan degradation to 2-amino-3- | Degradation/Utilization/Assimilation | AminoAcid |
|  | carboxymuconate semialdehyde |  |  |
| PWY-5652 | 2-amino-3-carboxymuconate semialdehyde | Degradation/Utilization/Assimilation | AminoAcid |
|  | degradation to glutaryl-CoA |  |  |
| PWY-5905 | hypusine biosynthesis | Biosynthesis | AminoAcid |
| PWY-5921 | L-glutamine tRNA biosynthesis | Biosynthesis | AminoAcid |
| PWY-6030 | serotonin and melatonin biosynthesis | Biosynthesis | AminoAcid |
| PWY-6100 | L-carnitine biosynthesis | Biosynthesis | AminoAcid |
| PWY-6117 | spermine and spermidine degradation I | Degradation/Utilization/Assimilation | AminoAcid |
| PWY-6133 | (S)-reticuline biosynthesis | Biosynthesis | AminoAcid |
| PWY-6158 | creatine-phosphate biosynthesis | Biosynthesis | AminoAcid |
| PWY-6173 | histamine biosynthesis | Biosynthesis | AminoAcid |
| PWY-6181 | histamine degradation | Degradation/Utilization/Assimilation | AminoAcid |
| PWY-6241 | thyroid hormone biosynthesis | Biosynthesis | AminoAcid |
| PWY-6260 | thyroid hormone metabolism I (via deiodination) | Degradation/Utilization/Assimilation | AminoAcid |
| PWY-6261 | thyroid hormone metabolism II (via conjugation | Degradation/Utilization/Assimilation | AminoAcid |
|  | and/or degradation) |  |  |
| PWY-6281 | selenocysteine biosynthesis | Biosynthesis | AminoAcid |
| PWY-6307 | tryptophan degradation X (mammalian, via | Degradation/Utilization/Assimilation | AminoAcid |
|  | tryptamine) |  |  |
| PWY-6313 | serotonin degradation | Degradation/Utilization/Assimilation | AminoAcid |
| PWY-6334 | L-dopa degradation | Degradation/Utilization/Assimilation | AminoAcid |
| PWY-6342 | noradrenaline and adrenaline degradation | Degradation/Utilization/Assimilation | AminoAcid |

| PWY-6481 | L-dopachrome biosynthesis | Biosynthesis | AminoAcid |
| --- | --- | --- | --- |
| PWY-6482 | diphthamide biosynthesis | Biosynthesis | AminoAcid |
| PWY-6498 | eumelanin biosynthesis | Biosynthesis | AminoAcid |
| PWY66-301 | catecholamine biosynthesis | Biosynthesis | AminoAcid |
| PWY66-425 | lysine degradation II (pipecolate pathway) | Degradation/Utilization/Assimilation | AminoAcid |
| PWY66-426 | hydrogen sulfide biosynthesis (trans-sulfuration) | Metabolic Clusters | AminoAcid |
| PWY66-428 | threonine degradation | Degradation/Utilization/Assimilation | AminoAcid |
| PWY6666-2 | dopamine degradation | Degradation/Utilization/Assimilation | AminoAcid |
| PWY-6688 | thyronamine and iodothyronamine metabolism | Degradation/Utilization/Assimilation | AminoAcid |
| PWY-6755 | S-methyl-5-thio-&alpha;-D-ribose 1- | Degradation/Utilization/Assimilation | AminoAcid |
|  | phosphate degradation |  |  |
| PWY-6756 | S-methyl-5'-thioadenosine degradation | Degradation/Utilization/Assimilation | AminoAcid |
| SAM-PWY | S-adenosyl-L-methionine biosynthesis | Biosynthesis | AminoAcid |
| SERDEG-PWY | L-serine degradation | Degradation/Utilization/Assimilation | AminoAcid |
| SER-GLYSYN-PWY-1 | serine and glycine biosynthesis | Superpathways | AminoAcid |
| SERSYN-PWY | serine biosynthesis (phosphorylated route) | Biosynthesis | AminoAcid |
| TRYPTOPHAN-DEGRADATION-1 | tryptophan degradation | Degradation/Utilization/Assimilation | AminoAcid |
| TYRFUMCAT-PWY | tyrosine degradation | Degradation/Utilization/Assimilation | AminoAcid |
| VALDEG-PWY | valine degradation | Degradation/Utilization/Assimilation | AminoAcid |
| PWY66-414 | superpathway of choline degradation to L-serine | Superpathways | AminoAcid |
| PWY-6292 | cysteine biosynthesis | Superpathways | AminoAcid |
| PWY66-401 | superpathway of tryptophan utilization | Superpathways | AminoAcid |
| COA-PWY-1 | coenzyme A biosynthesis | Biosynthesis | Cofactor |
| GLYCGREAT-PWY | creatine biosynthesis | Degradation/Utilization/Assimilation | Cofactor |
| HEME-BIOSYNTHESIS-II | heme biosynthesis from uroporphyrinogen-III I | Biosynthesis | Cofactor |
| NAD-BIOSYNTHESIS-III | NAD salvage | Biosynthesis | Cofactor |
| NADPHOS-DEPHOS-PWY-1 | NAD phosphorylation and dephosphorylation | Biosynthesis | Cofactor |
| NADSYN-PWY | NAD de novo biosynthesis | Biosynthesis | Cofactor |
| PLPSAL-PWY | pyridoxal 5'-phosphate salvage | Biosynthesis | Cofactor |
| PWY0-1264 | biotin-carboxyl carrier protein assembly | Biosynthesis | Cofactor |
| PWY0-1275 | lipoate biosynthesis and incorporation | Biosynthesis | Cofactor |
| PWY0-522 | lipoate salvage | Biosynthesis | Cofactor |
| PWY-2161 | folate polyglutamylation | Biosynthesis | Cofactor |
| PWY-2161B | glutamate removal from folates | Biosynthesis | Cofactor |
| PWY-2201 | folate transformations | Biosynthesis | Cofactor |
| PWY-5189 | tetrapyrrole biosynthesis | Biosynthesis | Cofactor |
| PWY-5653 | NAD biosynthesis from 2-amino-3- | Biosynthesis | Cofactor |
|  | carboxymuconate semialdehyde |  |  |

| PWY-5663 | tetrahydrobiopterin de novo biosynthesis | Biosynthesis | Cofactor |
| --- | --- | --- | --- |
| PWY-5754 | 4-hydroxybenzoate biosynthesis | Biosynthesis | Cofactor |
| PWY-5872 | ubiquinol-10 biosynthesis | Biosynthesis | Cofactor |
| PWY-5874 | heme degradation | Degradation/Utilization/Assimilation | Cofactor |
| PWY-5963 | thio-molybdenum cofactor biosynthesis | Biosynthesis | Cofactor |
| PWY-6076 | 1,25-dihydroxyvitamin D<sub>3</sub> | Biosynthesis | Cofactor |
|  | biosynthesis |  |  |
| PWY-6309 | L-kynurenine degradation | Superpathways | Cofactor |
| PWY-6430 | thymine degradation | Degradation/Utilization/Assimilation | Cofactor |
| PWY-6613 | tetrahydrofolate salvage from 5,10- | Biosynthesis | Cofactor |
|  | methenyltetrahydrofolate |  |  |
| PWY66-201 | nicotine degradation IV | Degradation/Utilization/Assimilation | Cofactor |
| PWY66-221 | nicotine degradation III | Degradation/Utilization/Assimilation | Cofactor |
| PWY66-366 | flavin biosynthesis | Biosynthesis | Cofactor |
| PWY-6823 | molybdenum cofactor biosynthesis | Biosynthesis | Cofactor |
| PWY-6857 | retinol biosynthesis | Biosynthesis | Cofactor |
| PWY-6872 | retinoate biosynthesis I | Biosynthesis | Cofactor |
| PWY-6875 | retinoate biosynthesis II | Biosynthesis | Cofactor |
| PWY-6898 | thiamin salvage III | Biosynthesis | Cofactor |
| PWY-6938 | NADH repair | Generation of Precursor Metabolites | Cofactor |
|  |  | and Energy |  |
| PWY-7250 | iron-sulfur cluster biosynthesis | Biosynthesis | Cofactor |
| PWY-7283 | wybutosine biosynthesis | Superpathways | Cofactor |
| PWY-7286 | 7-(3-amino-3-carboxypropyl)-wyosine biosynthesis Biosynthesis | | Cofactor |
| THIOREDOX-PWY | thioredoxin pathway | Biosynthesis | Cofactor |
| PWY-5920 | heme biosynthesis | Superpathways | Cofactor |
| DETOX1-PWY | superoxide radicals degradation | Detoxification | Detoxification |
| GLUT-REDOX-PWY | glutathione redox reactions II | Biosynthesis | Detoxification |
| MGLDLCTANA-PWY | methylglyoxal degradation VI | Detoxification | Detoxification |
| PWY-1801 | formaldehyde oxidation | Degradation/Utilization/Assimilation | Detoxification |
| PWY-4202 | arsenate detoxification I (glutaredoxin) | Detoxification | Detoxification |
| PWY-5386 | methylglyoxal degradation I | Detoxification | Detoxification |
| PWY-5453 | methylglyoxal degradation III | Detoxification | Detoxification |
| PWY-6502 | oxidized GTP and dGTP detoxification | Metabolic Clusters | Detoxification |
| PWY66-241 | bupropion degradation | Degradation/Utilization/Assimilation | Detoxification |
| PWY-7112 | 4-hydroxy-2-nonenal detoxification | Detoxification | Detoxification |
| PWY66-392 | lipoxin biosynthesis | Biosynthesis | FattyAcid/hormone |
| PWY66-393 | aspirin-triggered lipoxin biosynthesis | Biosynthesis | FattyAcid/hormone |

| PWY66-394 | aspirin triggered resolvin E biosynthesis | Biosynthesis | FattyAcid/hormone |
| --- | --- | --- | --- |
| PWY66-395 | aspirin triggered resolvin D biosynthesis | Biosynthesis | FattyAcid/hormone |
| PWY66-397 | resolvin D biosynthesis | Biosynthesis | FattyAcid/hormone |
| FAO-PWY | fatty acid &beta;-oxidation | Degradation/Utilization/Assimilation | FattyAcid/TAG |
| FASYN-ELONG-PWY | fatty acid elongation -- saturated | Biosynthesis | FattyAcid/TAG |
| LIPAS-PWY | triacylglycerol degradation | Degradation/Utilization/Assimilation | FattyAcid/TAG |
| LIPASYN-PWY | phospholipases | Metabolic Clusters | FattyAcid/TAG |
| PROPIONMET-PWY | propionyl-CoA degradation | Degradation/Utilization/Assimilation | FattyAcid/TAG |
| PWY-5130 | 2-oxobutanoate degradation | Superpathways | FattyAcid/TAG |
| PWY-5137 | fatty acid &beta;-oxidation (unsaturated, odd | Degradation/Utilization/Assimilation | FattyAcid/TAG |
|  | number) |  |  |
| PWY-5143 | fatty acid activation | Activation/Inactivation/Interconversion | FattyAcid/TAG |
| PWY-5148 | acyl-CoA hydrolysis | Biosynthesis | FattyAcid/TAG |
| PWY-5451 | acetone degradation I (to methylglyoxal) | Degradation/Utilization/Assimilation | FattyAcid/TAG |
| PWY-5966-1 | fatty acid biosynthesis initiation | Biosynthesis | FattyAcid/TAG |
| PWY-5972 | stearate biosynthesis | Biosynthesis | FattyAcid/TAG |
| PWY-5994 | palmitate biosynthesis | Biosynthesis | FattyAcid/TAG |
| PWY-5996 | oleate biosynthesis | Biosynthesis | FattyAcid/TAG |
| PWY-6000 | &gamma;-linolenate biosynthesis | Biosynthesis | FattyAcid/TAG |
| PWY-6012-1 | acyl carrier protein metabolism | Biosynthesis | FattyAcid/TAG |
| PWY-6111 | mitochondrial L-carnitine shuttle | Degradation/Utilization/Assimilation | FattyAcid/TAG |
| PWY-6535 | 4-aminobutyrate degradation | Degradation/Utilization/Assimilation | FattyAcid/TAG |
| PWY66-161 | oxidative ethanol degradation III | Degradation/Utilization/Assimilation | FattyAcid/TAG |
| PWY66-162 | ethanol degradation IV | Degradation/Utilization/Assimilation | FattyAcid/TAG |
| PWY66-21 | ethanol degradation II | Degradation/Utilization/Assimilation | FattyAcid/TAG |
| PWY66-374 | C20 prostanoid biosynthesis | Biosynthesis | FattyAcid/TAG |
| PWY66-375 | leukotriene biosynthesis | Biosynthesis | FattyAcid/TAG |
| PWY66-387 | fatty acid &alpha;-oxidation | Degradation/Utilization/Assimilation | FattyAcid/TAG |
| PWY66-388 | fatty acid &alpha;-oxidation III | Degradation/Utilization/Assimilation | FattyAcid/TAG |
| PWY66-389 | phytol degradation | Degradation/Utilization/Assimilation | FattyAcid/TAG |
| PWY66-391 | fatty acid &beta;-oxidation (peroxisome) | Degradation/Utilization/Assimilation | FattyAcid/TAG |
| PWY6666-1 | anandamide degradation | Degradation/Utilization/Assimilation | FattyAcid/TAG |
| PWY-7049 | eicosapentaenoate biosynthesis | Biosynthesis | FattyAcid/TAG |
| TRIGLSYN-PWY | triacylglycerol biosynthesis | Biosynthesis | FattyAcid/TAG |
| MALATE-ASPARTATE-SHUTTLE-PWY | malate-aspartate shuttle | Degradation/Utilization/Assimilation | Glycolysis/TCA/PentoseP |
| NONOXIPENT-PWY | pentose phosphate pathway (non-oxidative | Generation of Precursor Metabolites | Glycolysis/TCA/PentoseP |
|  | branch) | and Energy |  |

| OXIDATIVEPENT-PWY-1 | pentose phosphate pathway (oxidative branch) | Generation of Precursor Metabolites | Glycolysis/TCA/PentoseP |
| --- | --- | --- | --- |
|  |  | and Energy |  |
| PWY0-1313 | acetate conversion to acetyl-CoA | Degradation/Utilization/Assimilation | Glycolysis/TCA/PentoseP |
| PWY0-662 | PRPP biosynthesis | Biosynthesis | Glycolysis/TCA/PentoseP |
| PWY-4261 | glycerol degradation | Degradation/Utilization/Assimilation | Glycolysis/TCA/PentoseP |
| PWY-5084 | 2-oxoglutarate decarboxylation to succinyl-CoA | Degradation/Utilization/Assimilation | Glycolysis/TCA/PentoseP |
| PWY-5172 | acetyl-CoA biosynthesis from citrate | Generation of Precursor Metabolites | Glycolysis/TCA/PentoseP |
|  |  | and Energy |  |
| PWY-5481 | lactate fermentation (reoxidation of cytosolic | Generation of Precursor Metabolites | Glycolysis/TCA/PentoseP |
|  | NADH) | and Energy |  |
| PWY-6118 | glycerol-3-phosphate shuttle | Generation of Precursor Metabolites | Glycolysis/TCA/PentoseP |
|  |  | and Energy |  |
| PWY-6405 | Rapoport-Luebering glycolytic shunt | Biosynthesis | Glycolysis/TCA/PentoseP |
| PWY66-367 | ketogenesis | Generation of Precursor Metabolites | Glycolysis/TCA/PentoseP |
|  |  | and Energy |  |
| PWY66-368 | ketolysis | Generation of Precursor Metabolites | Glycolysis/TCA/PentoseP |
|  |  | and Energy |  |
| PWY66-398 | TCA cycle | Generation of Precursor Metabolites | Glycolysis/TCA/PentoseP |
|  |  | and Energy |  |
| PWY66-399 | gluconeogenesis | Biosynthesis | Glycolysis/TCA/PentoseP |
| PWY66-400 | glycolysis | Generation of Precursor Metabolites | Glycolysis/TCA/PentoseP |
|  |  | and Energy |  |
| PWY66-423 | fructose 2,6-bisphosphate | Biosynthesis | Glycolysis/TCA/PentoseP |
|  | synthesis/dephosphorylation |  |  |
| PYRUVDEHYD-PWY | pyruvate decarboxylation to acetyl CoA | Generation of Precursor Metabolites | Glycolysis/TCA/PentoseP |
|  |  | and Energy |  |
| PENTOSE-P-PWY | pentose phosphate pathway | Superpathways | Glycolysis/TCA/PentoseP |
| PWY66-407 | superpathway of conversion of glucose to acetyl | Superpathways | Glycolysis/TCA/PentoseP |
|  | CoA and entry into the TCA cycle |  |  |
| CHOLINE-BETAINE-ANA-PWY | choline degradation | Degradation/Utilization/Assimilation | MembraneLipids |
| MANNOSYL-CHITO-DOLICHOL- | dolichyl-diphosphooligosaccharide biosynthesis | Biosynthesis | MembraneLipids |
| BIOSYNTHESIS |  |  |  |
| PWY-2301 | myo-inositol de novo biosynthesis | Biosynthesis | MembraneLipids |
| PWY3DJ-11281 | sphingomyelin metabolism/ceramide salvage | Biosynthesis | MembraneLipids |
| PWY3DJ-11470 | sphingosine and sphingosine-1-phosphate | Degradation/Utilization/Assimilation | MembraneLipids |
|  | metabolism |  |  |
| PWY3DJ-12 | ceramide de novo biosynthesis | Biosynthesis | MembraneLipids |
| PWY3O-450 | phosphatidylcholine biosynthesis | Biosynthesis | MembraneLipids |
| PWY4FS-6 | phosphatidylethanolamine biosynthesis II | Biosynthesis | MembraneLipids |

| PWY-5269 | cardiolipin biosynthesis | Biosynthesis | MembraneLipids |
| --- | --- | --- | --- |
| PWY-5667 | CDP-diacylglycerol biosynthesis | Biosynthesis | MembraneLipids |
| PWY-6129 | dolichol and dolichyl phosphate biosynthesis | Biosynthesis | MembraneLipids |
| PWY-6351 | D-myo-inositol (1,4,5)-trisphosphate | Biosynthesis | MembraneLipids |
|  | biosynthesis |  |  |
| PWY-6352 | 3-phosphoinositide biosynthesis | Biosynthesis | MembraneLipids |
| PWY-6362 | 1D-myo-inositol hexakisphosphate | Biosynthesis | MembraneLipids |
|  | biosynthesis II (mammalian) |  |  |
| PWY-6363 | D-myo-inositol (1,4,5)-trisphosphate | Biosynthesis | MembraneLipids |
|  | degradation |  |  |
| PWY-6364 | D-myo-inositol (1,3,4)-trisphosphate | Biosynthesis | MembraneLipids |
|  | biosynthesis |  |  |
| PWY-6365 | D-myo-inositol (3,4,5,6)-tetrakisphosphate | Biosynthesis | MembraneLipids |
|  | biosynthesis |  |  |
| PWY-6366 | D-myo-inositol (1,4,5,6)-tetrakisphosphate | Biosynthesis | MembraneLipids |
|  | biosynthesis |  |  |
| PWY-6367 | D-myo-inositol-5-phosphate metabolism | Biosynthesis | MembraneLipids |
| PWY-6368 | 3-phosphoinositide degradation | Degradation/Utilization/Assimilation | MembraneLipids |
| PWY-6369 | inositol pyrophosphates biosynthesis | Biosynthesis | MembraneLipids |
| PWY-6554 | 1D-myo-inositol hexakisphosphate | Biosynthesis | MembraneLipids |
|  | biosynthesis V (from Ins(1,3,4)P3) |  |  |
| PWY-7501 | phosphatidylserine biosynthesis I | Biosynthesis | MembraneLipids |
| PWY-6358 | superpathway of D-myo-inositol (1,4,5)- | Superpathways | MembraneLipids |
|  | trisphosphate metabolism |  |  |
| PWY-6371 | superpathway of inositol phosphate compounds | Superpathways | MembraneLipids |
| P121-PWY | adenine and adenosine salvage I | Biosynthesis | Nucleotide |
| PWY0-1295 | pyrimidine ribonucleosides degradation | Degradation/Utilization/Assimilation | Nucleotide |
| PWY0-1296 | purine ribonucleosides degradation to ribose-1- | Degradation/Utilization/Assimilation | Nucleotide |
|  | phosphate |  |  |
| PWY-3982 | uracil degradation | Biosynthesis | Nucleotide |
| PWY-5686 | UMP biosynthesis | Biosynthesis | Nucleotide |
| PWY-5695 | urate biosynthesis/inosine 5'-phosphate | Degradation/Utilization/Assimilation | Nucleotide |
|  | degradation |  |  |
| PWY-6121 | 5-aminoimidazole ribonucleotide biosynthesis | Biosynthesis | Nucleotide |
| PWY-6124 | inosine-5'-phosphate biosynthesis | Biosynthesis | Nucleotide |
| PWY-6608 | guanosine nucleotides degradation | Degradation/Utilization/Assimilation | Nucleotide |
| PWY-6609 | adenine and adenosine salvage III | Biosynthesis | Nucleotide |
| PWY-6619 | adenine and adenosine salvage II | Biosynthesis | Nucleotide |
| PWY-6620 | guanine and guanosine salvage | Biosynthesis | Nucleotide |

| PWY66-385 | dTMP de novo biosynthesis (mitochondrial) | Biosynthesis | Nucleotide |
| --- | --- | --- | --- |
|  |  |  |  |
| PWY66-420 | carnosine biosynthesis | Biosynthesis | Nucleotide |
| PWY66-421 | homocarnosine biosynthesis | Biosynthesis | Nucleotide |
| PWY-7176 | UTP and CTP de novo biosynthesis | Biosynthesis | Nucleotide |
| PWY-7177 | UTP and CTP dephosphorylation II | Degradation/Utilization/Assimilation | Nucleotide |
| PWY-7179-1 | purine deoxyribonucleosides degradation | Degradation/Utilization/Assimilation | Nucleotide |
| PWY-7180 | 2'-deoxy-&alpha;-D-ribose 1-phosphate | Degradation/Utilization/Assimilation | Nucleotide |
|  | degradation |  |  |
| PWY-7181 | pyrimidine deoxyribonucleosides degradation | Degradation/Utilization/Assimilation | Nucleotide |
| PWY-7184 | pyrimidine deoxyribonucleotides de novo | Metabolic Clusters | Nucleotide |
|  | biosynthesis |  |  |
| PWY-7185 | UTP and CTP dephosphorylation I | Degradation/Utilization/Assimilation | Nucleotide |
| PWY-7193 | pyrimidine ribonucleosides salvage I | Biosynthesis | Nucleotide |
| PWY-7197 | pyrimidine deoxyribonucleotide phosphorylation | Metabolic Clusters | Nucleotide |
| PWY-7199 | pyrimidine deoxyribonucleosides salvage | Biosynthesis | Nucleotide |
| PWY-7205 | CMP phosphorylation | Biosynthesis | Nucleotide |
| PWY-7210 | pyrimidine deoxyribonucleotides biosynthesis from Metabolic Clusters | | Nucleotide |
|  | CTP |  |  |
| PWY-7219 | adenosine ribonucleotides de novo | Biosynthesis | Nucleotide |
|  | biosynthesis |  |  |
| PWY-7221 | guanosine ribonucleotides de novo | Biosynthesis | Nucleotide |
|  | biosynthesis |  |  |
| PWY-7224 | purine deoxyribonucleosides salvage | Metabolic Clusters | Nucleotide |
| PWY-7226 | guanosine deoxyribonucleotides de novo | Biosynthesis | Nucleotide |
|  | biosynthesis |  |  |
| PWY-7227 | adenosine deoxyribonucleotides de novo | Biosynthesis | Nucleotide |
|  | biosynthesis |  |  |
| SALVADEHYPOX-PWY | adenosine nucleotides degradation | Degradation/Utilization/Assimilation | Nucleotide |
| PWY0-162 | superpathway of pyrimidine ribonucleotides de | Superpathways | Nucleotide |
|  | novo biosynthesis |  |  |
| PWY-841 | purine nucleotides de novo biosynthesis | Superpathways | Nucleotide |
| PWY-6353 | purine nucleotides degradation | Superpathways | Nucleotide |
| PWY-7209 | pyrimidine ribonucleosides degradation | Superpathways | Nucleotide |
| PWY-7200 | superpathway of pyrimidine deoxyribonucleoside | Superpathways | Nucleotide |
|  | salvage |  |  |
| PWY-7228 | guanosine nucleotides de novo | Superpathways | Nucleotide |
|  | biosynthesis |  |  |

| PWY-7211 | superpathway of pyrimidine deoxyribonucleotides | Superpathways | Nucleotide |
| --- | --- | --- | --- |
|  | de novo biosynthesis |  |  |
| PWY66-409 | superpathway of purine nucleotide salvage | Superpathways | Nucleotide |
| BGALACT-PWY | lactose degradation III | Degradation/Utilization/Assimilation | Polysaccharides |
| GLUAMCAT-PWY | N-acetylglucosamine degradation I | Degradation/Utilization/Assimilation | Polysaccharides |
| MANNCAT-PWY | D-mannose degradation | Degradation/Utilization/Assimilation | Polysaccharides |
| PWY0-1182 | trehalose degradation | Degradation/Utilization/Assimilation | Polysaccharides |
| PWY-4101 | sorbitol degradation I | Degradation/Utilization/Assimilation | Polysaccharides |
| PWY-4821 | UDP-D-xylose and UDP-D-glucuronate | Biosynthesis | Polysaccharides |
|  | biosynthesis |  |  |
| PWY-5067 | glycogen biosynthesis | Biosynthesis | Polysaccharides |
| PWY-5512 | UDP-N-acetyl-D-galactosamine | Biosynthesis | Polysaccharides |
|  | biosynthesis I |  |  |
| PWY-5514 | UDP-N-acetyl-D-galactosamine | Biosynthesis | Polysaccharides |
|  | biosynthesis II |  |  |
| PWY-55y5 | D-glucuronate degradation | Degradation/Utilization/Assimilation | Polysaccharides |
| PWY-5659 | GDP-mannose biosynthesis | Biosynthesis | Polysaccharides |
| PWY-5661-1 | GDP-glucose biosynthesis II | Biosynthesis | Polysaccharides |
| PWY-5941-1 | glycogenolysis | Degradation/Utilization/Assimilation | Polysaccharides |
| PWY-6 | GDP-L-fucose biosynthesis II (from L-fucose) | Biosynthesis | Polysaccharides |
| PWY-6138 | CMP-N-acetylneuraminate biosynthesis I | Biosynthesis | Polysaccharides |
|  | (eukaryotes) |  |  |
| PWY-6517 | N-acetylglucosamine degradation II | Superpathways | Polysaccharides |
| PWY-6558 | heparan sulfate biosynthesis (late stages) | Biosynthesis | Polysaccharides |
| PWY-6566 | chondroitin and dermatan biosynthesis | Biosynthesis | Polysaccharides |
| PWY-6567 | chondroitin sulfate biosynthesis (late stages) | Biosynthesis | Polysaccharides |
| PWY-6568 | dermatan sulfate biosynthesis (late stages) | Biosynthesis | Polysaccharides |
| PWY-6573 | chondroitin sulfate degradation (metazoa) | Degradation/Utilization/Assimilation | Polysaccharides |
| PWY-6576 | dermatan sulfate degradation (metazoa) | Degradation/Utilization/Assimilation | Polysaccharides |
| PWY-66 | GDP-L-fucose biosynthesis I (from GDP-D- | Biosynthesis | Polysaccharides |
|  | mannose) |  |  |
| PWY66-373 | sucrose degradation | Degradation/Utilization/Assimilation | Polysaccharides |
| PWY66-422 | D-galactose degradation V (Leloir pathway) | Degradation/Utilization/Assimilation | Polysaccharides |
| UDPNACETYLGALSYN-PWY | UDP-N-acetyl-D-glucosamine biosynthesis | Biosynthesis | Polysaccharides |
|  | II |  |  |
| PWY-6569 | chondroitin sulfate biosynthesis | Superpathways | Polysaccharides |
| PWY-6571 | dermatan sulfate biosynthesis | Superpathways | Polysaccharides |
| PWY-5525 | D-Glucuronate-Degradation | Degradation/Utilization/Assimilation | Polysaccharides |

| PWY-6398 | melatonin degradation I | Degradation/Utilization/Assimilation | SecondaryMetabolism |
| --- | --- | --- | --- |
| PWY-6399 | melatonin degradation II | Degradation/Utilization/Assimilation | SecondaryMetabolism |
|  | superpathway of geranylgeranyldiphosphate | Superpathways | SecondaryMetabolism |
|  | biosynthesis I (via mevalonate) |  |  |
| PWY-6402 | superpathway of melatonin degradation | Superpathways | SecondaryMetabolism |
| PWY-5120 | geranylgeranyldiphosphate biosynthesis | Biosynthesis | Steroid |
| PWY-5123 | trans, trans-farnesyl diphosphate | Biosynthesis | Steroid |
|  | biosynthesis |  |  |
| PWY-5670 | epoxysqualene biosynthesis | Biosynthesis | Steroid |
| PWY-6061 | bile acid biosynthesis, neutral pathway | Biosynthesis | Steroid |
| PWY-6074 | zymosterol biosynthesis | Biosynthesis | Steroid |
| PWY-6132 | lanosterol biosynthesis | Biosynthesis | Steroid |
| PWY-6377 | &alpha;-tocopherol degradation | Degradation/Utilization/Assimilation | Steroid |
| PWY66-3 | cholesterol biosynthesis II (via 24,25- | Superpathways | Steroid |
|  | dihydrolanosterol) |  |  |
| PWY66-341 | cholesterol biosynthesis I | Superpathways | Steroid |
| PWY66-377 | pregnenolone biosynthesis | Biosynthesis | Steroid |
| PWY66-378 | androgen biosynthesis | Biosynthesis | Steroid |
| PWY66-380 | estradiol biosynthesis I | Biosynthesis | Steroid |
| PWY66-381 | glucocorticoid biosynthesis | Biosynthesis | Steroid |
| PWY66-382 | mineralocorticoid biosynthesis | Biosynthesis | Steroid |
| PWY66-4 | cholesterol biosynthesis III (via desmosterol) | Superpathways | Steroid |
| PWY-7299 | progesterone biosynthesis | Biosynthesis | Steroid |
| PWY-7306 | estradiol biosynthesis II | Biosynthesis | Steroid |
| PWY-7455 | allopregnanolone biosynthesis | Biosynthesis | Steroid |
| PWY-922 | mevalonate pathway | Biosynthesis | Steroid |
| PWY-7305 | superpathway of steroid hormone biosynthesis | Superpathways | Steroid |
| PWY66-5 | superpathway of cholesterol biosynthesis | Superpathways | Steroid |
