# Supplementary material for: Influence of pathway topology and functional class on the molecular evolution of human metabolic genes
Source: PLoS One. 2018 Dec 14;13(12):e0208782. doi: 10.1371/journal.pone.0208782 (PMC6294346; doi:10.1371/journal.pone.0208782)
Supplement: S1 Fig — (DOCX) [file pone.0208782.s002.docx]

| 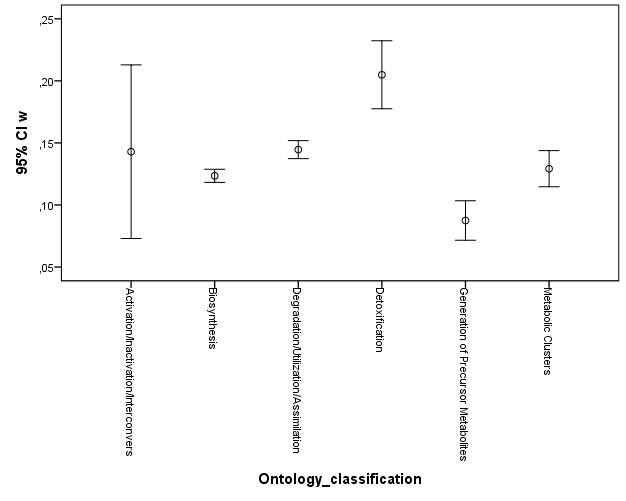 | 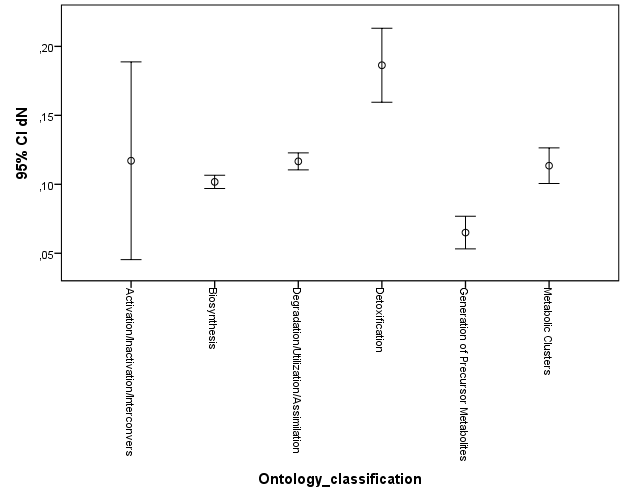 | 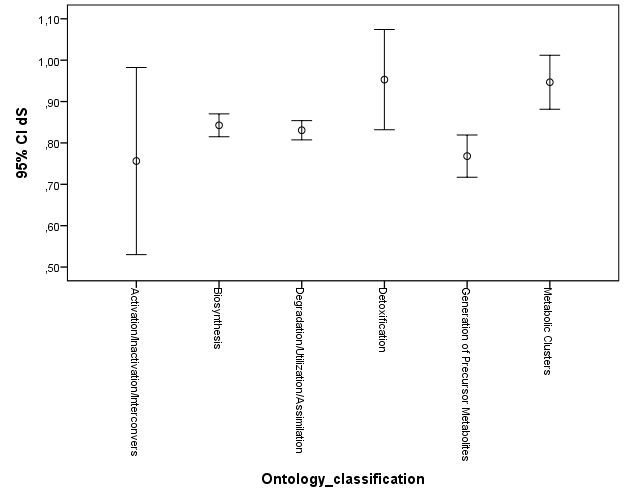 |
| --- | --- | --- |

**S1 Fig.** **Graph representing *dN/dS*, *dN* and *dS* among genes belonging to different functional classes according to ontology-based classification.**
