# Supplementary material for: Influence of pathway topology and functional class on the molecular evolution of human metabolic genes
Source: PLoS One. 2018 Dec 14;13(12):e0208782. doi: 10.1371/journal.pone.0208782 (PMC6294346; doi:10.1371/journal.pone.0208782)
Supplement: S3 Fig — (DOCX) [file pone.0208782.s004.docx]

| 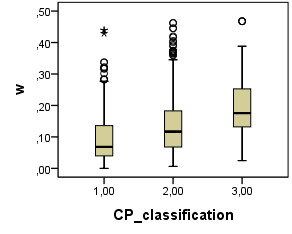 | 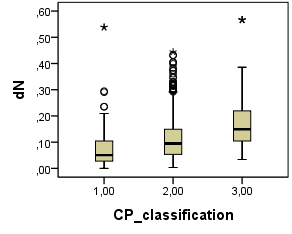 | 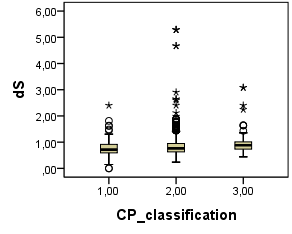 |
| --- | --- | --- |

**S3 Fig. Boxplots representing ω (*dN/dS)*, *dN* and *dS* among genes belonging to different functional classes.** Class 1 comprises the inner metabolism (Glycosis/TCA/PentoseP, Polysaccharides), class 2 comprises the second layer (Membrane Lipids metabolism, nucleotide metabolism, Fatty acid/TAG, Cofactor, Fatty Acid/hormone, and Aminoacid) while class 3 comprises the outer layer of cell metabolism (Steroid, Secondary Metabolism and Detoxification).. Boxes are 25th and 75th quartiles, black bar within the box represents the median, whiskers indicate minimum and maximum and dots and stars represent most extreme data point higher than 1.5 interquartile range from box.
