# Supplementary material for: Influence of pathway topology and functional class on the molecular evolution of human metabolic genes
Source: PLoS One. 2018 Dec 14;13(12):e0208782. doi: 10.1371/journal.pone.0208782 (PMC6294346; doi:10.1371/journal.pone.0208782)
Supplement: S4 Fig — (DOCX) [file pone.0208782.s005.docx]

a)

| 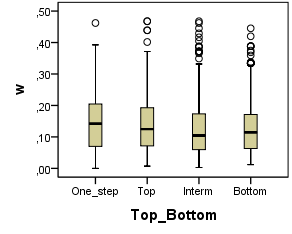 | 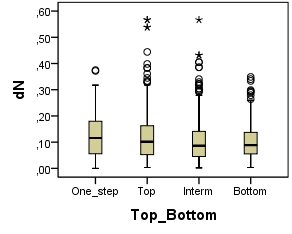 | 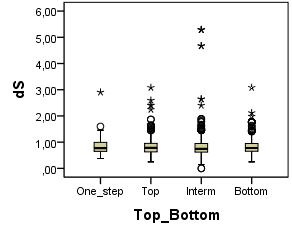 |
| --- | --- | --- |

b)

| 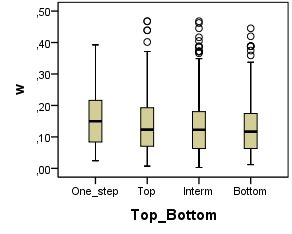 | 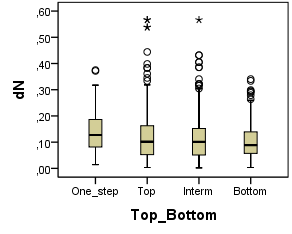 | 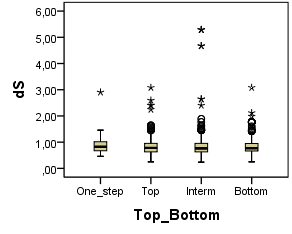 |
| --- | --- | --- |

**S4 Fig. Boxplots representing ω (dN/dS), dN and dS among genes according to the position in the pathway. a)** Boxplots representing *dN/dS*, *dN* and *dS* among genes whose position within the pathway belongs to four classes (one-step, top, intermediate, bottom positions) for the 275 base pathways. **b)** The same for the 208 base pathways with no loops. Dots show the mean ± 2 standard error (SE). Boxes are 25th and 75th quartiles, black bar within the box represents the median, whiskers indicate minimum and maximum and dots and stars represent most extreme data point higher than 1.5 interquartile range from the box.
